# Coreceptors and TCR Signaling – the Strong and the Weak of It

**DOI:** 10.3389/fcell.2020.597627

**Published:** 2020-10-16

**Authors:** Alexander M. Mørch, Štefan Bálint, Ana Mafalda Santos, Simon J. Davis, Michael L. Dustin

**Affiliations:** ^1^The Kennedy Institute of Rheumatology, University of Oxford, Oxford, United Kingdom; ^2^Radcliffe Department of Medicine, John Radcliffe Hospital, University of Oxford, Oxford, United Kingdom; ^3^MRC Human Immunology Unit, Weatherall Institute of Molecular Medicine, University of Oxford, Oxford, United Kingdom

**Keywords:** CD4, CD8, T-cell signaling, TCR triggering, antigen discrimination

## Abstract

The T-cell coreceptors CD4 and CD8 have well-characterized and essential roles in thymic development, but how they contribute to immune responses in the periphery is unclear. Coreceptors strengthen T-cell responses by many orders of magnitude – beyond a million-fold according to some estimates – but the mechanisms underlying these effects are still debated. T-cell receptor (TCR) triggering is initiated by the binding of the TCR to peptide-loaded major histocompatibility complex (pMHC) molecules on the surfaces of other cells. CD4 and CD8 are the only T-cell proteins that bind to the same pMHC ligand as the TCR, and can directly associate with the TCR-phosphorylating kinase Lck. At least three mechanisms have been proposed to explain how coreceptors so profoundly amplify TCR signaling: (1) the Lck recruitment model and (2) the pseudodimer model, both invoked to explain receptor triggering *per se*, and (3) two-step coreceptor recruitment to partially triggered TCRs leading to signal amplification. More recently it has been suggested that, in addition to initiating or augmenting TCR signaling, coreceptors effect antigen discrimination. But how can any of this be reconciled with TCR signaling occurring in the absence of CD4 or CD8, and with their interactions with pMHC being among the weakest specific protein-protein interactions ever described? Here, we review each theory of coreceptor function in light of the latest structural, biochemical, and functional data. We conclude that the oldest ideas are probably still the best, i.e., that their weak binding to MHC proteins and efficient association with Lck allow coreceptors to amplify weak incipient triggering of the TCR, without comprising TCR specificity.

## Introduction

Adaptive immune responses are initiated by T cells which continually patrol lymphoid and peripheral tissues for peptide, lipid or metabolite-derived antigens. Conventional T cells are activated through the binding of their αβ T-cell receptors (TCRs) to peptide-loaded major histocompatibility complex (pMHC) molecules on the surfaces of other cells. T-cell activation then leads to clonal expansion and the deployment of a battery of effector functions. T cells with distinct “helper” or “cytotoxic” activities were described as early as the 1960s and 70s (Bach et al., 1976). It was quickly established that these subsets could be distinguished by the mutually exclusive expression of just two cell surface markers, CD4 and CD8: CD4^+^ T cells aided antibody-producing B cells (Cantor and Boyse, 1977) while CD8^+^ T cells directly killed infected targets (Shiku et al., 1975). However, experiments with T cell clones and blocking antibodies by Swain and others showed that expression of these markers did not correlate fully with effector function (Swain, 1983). Instead, it appeared that they had a more exclusive role in determining which class of MHC molecule was being recognized. CD4 and CD8 were first referred to as “coreceptors” by Janeway (1988), distinguishing them from simple “accessory molecules” based on emerging evidence that they physically associated with the TCR complex during T-cell activation, and in recognition of their especially large effects on T-cell responses. Parnes et al. (1989) subsequently confirmed that CD4 and CD8 bind to MHC class II (MHC-II) and MHC class I (MHC-I), respectively. The discovery that CD4 and CD8 are both associated with the TCR-phosphorylating Src-family kinase Lck further heightened their special status (Veillette et al., 1988, 1989; Barber et al., 1989; Rudd et al., 1989; Zamoyska et al., 1989).

Coreceptors are known to have important roles in driving the thymic development of CD4^+^ or CD8^+^ T cells, in effect by signaling to thymocytes depending on whether their TCRs bind to MHC-II or MHC-I, respectively (Tikhonova et al., 2012). Precisely how they contribute to the functions of peripheral T cells is still debated, however. TCR phosphorylation (i.e., triggering) can be induced by high-affinity ligands in the absence of coreceptors, but CD4 and CD8 significantly augment antigen sensitivity and are essential for responding to some ligands (Janeway et al., 1988; Hampl et al., 1997; Holler and Kranz, 2003). Early proposals were that coreceptors either initiated signaling by recruiting Lck to the TCR (Rudd, 1990; Janeway, 1992) or amplified signaling by stabilizing the ternary complex (Xu and Littman, 1993). Another possibility was that coreceptors cross-link TCR-pMHC complexes to produce receptor dimers (Irvine et al., 2002). In addition to these direct effects on receptor signaling, serial “scanning” for the small subset of coreceptors that are stably associated with Lck has been invoked as a form of kinetic proofreading (Stepanek et al., 2014).

In this review, we start by providing a context for how coreceptors work by discussing how T cells come into contact with antigen and how this leads to intracellular signaling. We then discuss new insights into the structure and behavior of CD4 and CD8 and consider the present status of each of the models of coreceptor function. Finally, we consider the roles of CD4 and CD8 in thymic development and antigen discrimination.

## T-Cell Activation

### Microvilli and Microclusters

**FIGURE 1:**
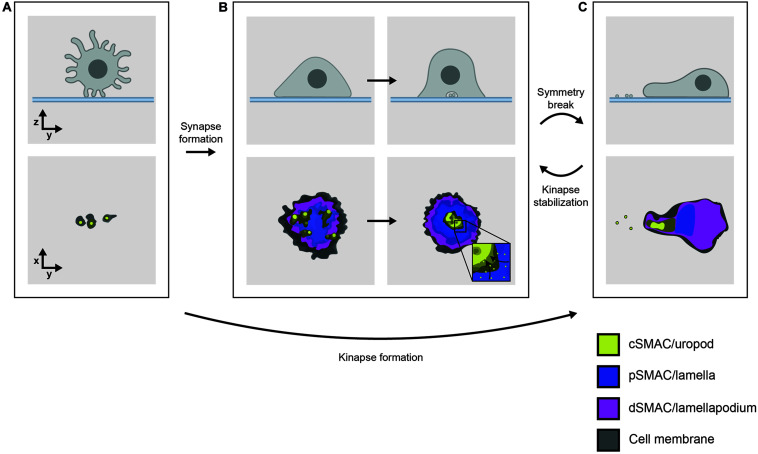
**(A)** T cells interact with functionalized lipid bilayers using multiple microvilli, forming either **(B)** a radially symmetric immunological synapse or **(C)** an asymmetrical, motile kinapse. These structures consist of organized SMAC domains which correspond to the underlying actin networks, indicated by color. Effector vesicles/particles are indicated by small membrane-bound circles. The kinapse is the primary behavior adopted by most human T cells stimulated by antigen with the exception of CD8^+^ memory T cells which are more likely to form stable synapses.

T cells need to approach antigen-presenting cells (APCs) within a distance of ∼15 nm for TCRs and coreceptors to interact with pMHC. This presents a challenge for two main reasons. First, T cells are highly motile lymphocytes that form only transient contacts with APCs (Miller et al., 2002; Mandl et al., 2012; Cai et al., 2017). Second, leukocyte surfaces are covered in a dense glycocalyx barrier which sterically hinders the formation of close cell-cell contacts (Springer, 1990). However, it is becoming clear that both thymocytes and T cells interact with neighboring cells using numerous small, febrile membrane projections called filopodia or microvilli (Figure 1A; Majstoravich et al., 2004), potentially in order to overcome these obstacles. The flexibility and dynamics of these F-actin-enriched structures seem well-suited to extensive and rapid exploration of the surfaces of other cells in the search for antigens (Cai et al., 2017).

The recognition of a cognate TCR ligand leads TCR-coreceptor-pMHC interactions to initiate inside-out signaling to integrin-family adhesion molecules, resulting in a dramatic increase in the contact area. Ligand-engaged TCRs nucleate submicron regions called microclusters where cytosolic signaling proteins also accumulate (Bunnell et al., 2002; Campi et al., 2005). Sustained TCR signaling leads to large scale re-organization of TCR-pMHC and adhesive interactions into a radially symmetric structure called the immunological synapse (Grakoui et al., 1999), which arrests cell motility and allows for the delivery of effector functions (Figure 1B). Immunofluorescence imaging of T cells interacting with B cells or planar antigen-presenting substrates showed how this synaptic interface takes on a characteristic “bull’s-eye” pattern of concentric rings referred to as supramolecular activation clusters (SMACs) (Monks et al., 1998). The canonical synapse consisted of three SMACs: the central (c)SMAC containing TCR/pMHC clusters, the peripheral (p)SMAC comprising adhesive LFA-1/ICAM-1 interactions, and the distal (d)SMAC marked by the presence of the large tyrosine phosphatase CD45 (Dustin et al., 1998; Monks et al., 1998; Freiberg et al., 2002).

This early view of the synapse has since been refined with advances in imaging technology revealing much about the complexity and dynamics of synapse formation. One principle that has emerged is that SMACs align with the different morphologies of the underlying F-actin networks; the dSMAC corresponds to a lamellipodium, the pSMAC to contractile lamella and the cSMAC to an F-actin-depleted secretory domain (Stinchcombe et al., 2001; Kaizuka et al., 2007; Yi et al., 2012; Fritzsche et al., 2017). Total internal reflection fluorescence microscopy, which selectively illuminates fluorophores close to the basal surface, has demonstrated that CD45 is relatively uniformly distributed across the synapse rather than being concentrated in the dSMAC as previously thought (Varma et al., 2006). The dSMAC also contains a substructure called the “corolla” which consists of sub-micron petal-like clusters of the CD2-CD58 adhesion pair and influences the distribution of important stimulatory/inhibitory molecules like CD28 and PD-1 (Demetriou et al., 2019). While TCR signaling was originally thought to be sustained in the cSMAC, it is now known to occur mainly in the dSMAC. TCRs are monovalent in their “resting” state, implying that the earliest signaling events are likely driven by this form of the complex (Brameshuber et al., 2018; Rossboth et al., 2018). Signaling TCRs then quickly form peripheral microclusters enriched in signaling proteins (Yokosuka et al., 2005) that perhaps enable sustained signaling. Microclusters frequently form at the tips of microvilli (Sage et al., 2012; Kumari et al., 2015; Jung et al., 2016) and migrate centripetally toward the cSMAC where signaling is eventually terminated (Figure 1B, inset; Varma et al., 2006). The cSMAC is now thought to comprise a complex vesicular sorting structure (Choudhuri et al., 2014), which secretes extracellular vesicles or particles that deliver effector functions across the synapse in both CD4^+^ (Saliba et al., 2019) and CD8^+^ T cells (Balint et al., 2020).

Symmetry breaking of synapses allows motile “kinapses” to form that sustain extensive areas of close contact during cell movement (Figure 1C; Dustin, 2007; Sims et al., 2007). The kinapse is the default behavior of most human T cells during antigenic stimulation (Mayya et al., 2018). The maintenance of a symmetrical synapse is only a characteristic of CD8^+^ effector T cells (Mayya et al., 2018) and appears to require WASP-dependent cytoskeletal tension (Sims et al., 2007; Kumari et al., 2020). TCR microclusters exhibit similar dynamics in synapses and kinapses, but remain stationary in the latter as the cell body moves past them, rather than moving centripetally (Beemiller et al., 2012).

The environments in which TCR- and coreceptor-binding to pMHC can initiate signaling thus include close contacts at microvillar tips, submicron microclusters within synapses/kinapses, and the much larger CD2 corolla, likely in that order. The organization and functions of each of these structures is a matter of intense investigation (Chang et al., 2016; Jung et al., 2016; Su et al., 2016; Demetriou et al., 2019). Although early imaging studies showed that CD4 and CD8 are recruited to the immunological synapse (Kupfer et al., 1987; Krummel et al., 2000; Zal et al., 2002), evidence for how they are organized within the smaller structures is only now beginning to emerge, including the suggestion that CD4 is pre-clustered at microvillar tips (Ghosh et al., 2020). But these environments are also highly dynamic, with remodeling on the order of seconds to minutes. Studies with a high spatial and temporal resolving power [e.g., imaging, spectroscopy and Förster resonance energy transfer (FRET) methods] will therefore be needed to understand the organization and evolution of TCR/pMHC/coreceptor interactions within these structures, and to understand how the very earliest stages of TCR signaling are influenced as a result.

### TCR Signaling

The αβ TCR is expressed at the T-cell surface as a signaling-competent assembly with three CD3 dimers (CD3γε, CD3δε, and CD247/CD3ζζ), the full structure of which was recently determined by cryo-electron microscopy (Dong et al., 2019). The α and β subunits of the TCR heterodimer are structurally similar and each consist of variable and constant extracellular protein domains, a transmembrane helix and short cytoplasmic tails lacking any folded structure or known function beyond stabilizing the heterodimer at the membrane. TCR signaling is initiated by the phosphorylation of immunoreceptor tyrosine-based activation motifs (ITAMs) located within the intracellular tails of the CD3 subunits by Src-family kinases Lck and Fyn. Phosphorylated ITAMs then serve as docking sites for the SH2 domains of ZAP-70, a kinase whose activation is enhanced by Lck phosphorylation. ZAP-70, in turn, phosphorylates adaptor proteins LAT and SLP-76 which form signaling “scaffolds” to which downstream signaling proteins such as phospholipase C (PLC)-γ are recruited. PLC-γ catalyzes the production of second messengers diacetyl glycerol and inositol triphosphate which increase cytoplasmic Ca^2+^ levels and activate Ras and protein kinase C, initiating more signaling that ultimately promotes cell proliferation and differentiation (Smith-Garvin et al., 2009; Hwang et al., 2020). But what produces TCR phosphorylation in the first instance? Several theories have been proposed and these are broadly categorized as oligomerization-, conformational change- or segregation-based models, which have been expertly reviewed elsewhere (van der Merwe and Dushek, 2011; Mariuzza et al., 2020). Given that CD4 and CD8 bind the same pMHC ligand as the TCR, and are the only proteins known to be constitutively attached, at least to some degree, to the Src kinase Lck, the coreceptors must contribute to the earliest signaling events. The questions are: at what stage and how?

## CD4 and CD8 – An Overview of Their Structure and Function

### Structure and Binding Properties

Like the TCR, CD4 and CD8 both belong to the immunoglobulin superfamily (IgSF) insofar as they each have extracellular IgSF domains that are attached to a transmembrane segment and a short cytoplasmic tail (Leahy, 1995). Uniquely among receptors expressed by lymphocytes, the cytoplasmic tails of both coreceptors contain motifs that associate with membrane-anchored Lck (Veillette et al., 1988; Barber et al., 1989). CD4 and CD8 have few other structural similarities, however, suggesting that coreceptors may need simply (1) to bind MHC proteins and (2) to associate stably with Lck in order to perform their functions. CD4 is a monomer with four concatenated extracellular V- and C-set IgSF domains whereas CD8 is typically expressed as a disulfide-linked heterodimer of α and β subunits each comprising single extracellular V-set domains perched on top of a heavily O-glycosylated “stalk” (Li et al., 2013). The cytoplasmic regions of both coreceptors also contain membrane-proximal cysteines that are post-translationally palmitoylated (Crise and Rose, 1992; Arcaro et al., 2000).

A number of immune cell lineages express an alternate homodimeric form of CD8 consisting only of α-subunits. CD8αα binds MHC-I with a similar affinity to CD8αβ (Kern et al., 1999; Leishman et al., 2001) but it cannot fully substitute for CD8αβ as a coreceptor in T cells (Gangadharan and Cheroutre, 2004). Since the α-subunit associates with Lck (Turner et al., 1990) and the β-subunit is palmitoylated (Arcaro et al., 2001), CD8αα could in principle associate with two Lck molecules. There is evidence, however, that CD8β enhances the association of Lck with CD8α (Bosselut et al., 2000) implying that coreceptor palmitoylation is important for this interaction. Whether this is through post-translational co-trafficking through the ER (Shaw et al., 1989) or partitioning into membrane domains (He and Marguet, 2008) is unclear.

Coreceptors bind to MHC proteins with exceptionally low affinities – the CD4/MHC-II affinity, in particular, is among the weakest measured for any pair of interacting proteins (Jönsson et al., 2016). Surface plasmon resonance (SPR) assays, which are ideal for detecting weak protein-protein interactions, place the solution (3D) K_d_ value for the CD8αα/MHC-I interaction at ∼200 μM at 37°C (Wyer et al., 1999). Although SPR measurements of CD8αβ/MHC-I binding have not yet been conducted at 37°C, assays at room temperature suggest that CD8αα and CD8αβ have similar affinities for MHC-I (Kern et al., 1999; Leishman et al., 2001). SPR-based assays have thus far failed to reveal binding between soluble forms of CD4 and MHC-II, suggesting a lower limit of 2.5 mM for the 3D K_d_ value at 37°C (Jönsson et al., 2016). However, interactions between cell surface proteins are largely constrained to a two-dimensional (2D) plane and are therefore better described with 2D K_d_ values (i.e., the density of counter-receptors at which 50% of the receptor is bound) (Bell et al., 1984; Dustin et al., 1996; Zhu et al., 2007). Although no 2D K_d_ has yet been reported for CD8/MHC-I interactions, the 2D K_d_ for CD4/MHC-II binding was measured to be ∼5,000 molecules/μm^2^ in a cell-bilayer contact using the rat CD2-CD48 adhesion pair to create a physiologically relevant membrane distance (Jönsson et al., 2016). One method of estimating the corresponding 3D K_d_ involves calculating a “confinement region” which takes into account the entropic and geometric constraints that arise from confining interactions to a plane (Dustin et al., 1997). The confinement region given by the 2D and 3D binding constants for CD2-CD48 produces a 3D K_d_ value of ∼5.1 mM for the CD4/MHC-II interaction, in agreement with the lower limit of 2.5 mM estimated using SPR (Jönsson et al., 2016). The remarkably low affinity of coreceptor/MHC binding has two important implications: (1) biologically important interactions may be undetectable using SPR assays, and (2) coreceptor/MHC interactions are unlikely to occur spontaneously at the cell surface (van der Merwe and Davis, 2003). Supporting the latter, biophysical studies show minimal binding of coreceptors to MHC, except in the presence of TCR (Huang et al., 2007; van der Merwe and Cordoba, 2011; Hong et al., 2015).

These very low affinities probably also explain why it took so long to crystallize the ternary TCR/pMHC/coreceptor complex. Indeed to produce crystals, Mariuzza et al. (2020) had to engineer an affinity-enhanced version of CD4 capable of forming a stable ternary complex (Yin et al., 2012). The structure revealed a distinctive V-shaped arch in which the TCR and CD4 are tilted ∼65° relative to the T-cell surface, apparently precluding any direct interaction between them. Once the complex had been solved it allowed the testing of various TCR triggering theories. On the basis of a dimerization site observed in the CD4 crystal lattice (Wu et al., 1997), CD4 homodimers have been proposed to contribute to T-cell activation through non-specific effects or by cross-linking MHC molecules to increase the avidity of TCR/pMHC binding (Moldovan et al., 2006). However, CD4 dimerization is incompatible with the geometry imposed by the ternary TCR/pMHC/CD4 structure (Yin et al., 2012). The suggestion that MHC proteins are functional dimers (Brown et al., 1993) is also problematic because the claimed MHC dimerization site overlaps with the CD4/MHC-II binding site (Yin et al., 2012). Finally, TCR dimers have also been proposed to explain triggering (Kuhns et al., 2010). Unlike CD4 and MHC dimerization, this model is feasible because the proposed TCR dimerization site is located on a contiguous surface on TCRα outside of the TCR-pMHC-CD4 arch (Yin et al., 2012). However, a survey of 22 other TCR structures did not find similar dimerization sites (Wang and Reinherz, 2013) and conserved glycans in this region are thought to sterically preclude dimerization (Li et al., 2013). These observations highlight the need for orthogonal *in situ* approaches to validate protein-protein interactions inferred from structural studies. Using two fluorescence-based approaches, James et al. (2011) showed that CD4-Lck molecules are monovalent at the surfaces of live cells implying that coreceptors are likely to be functionally monovalent.

The structure of the ternary complex also suggested that the coreceptor would be positioned adjacent to the CD3 chains, whose location at that stage was unclear (Yin et al., 2012). The recent determination of the TCR/CD3 complex by Dong et al. (2019) confirms this arrangement, as a model made by superimposing the TCR/CD3 structure with the ternary complex shows that the CD3 chains are placed in the middle of the “arch,” ideally positioned to be phosphorylated by CD4-Lck. Altogether, the structural data indicate that there is no contact between the coreceptor and the CD3 chain ectodomains, making it unlikely that the recruitment of CD4 or CD8 is directly enhanced by interactions involving their extracellular regions. As we discuss below, this makes their recruitment likely to be secondary to TCR triggering.

The binding of coreceptors to MHC in the absence of the TCR is proposed be a general mechanism of increasing T-cell/APC adhesion (Glatzová and Cebecauer, 2019). This, however, seems incompatible with their extremely low affinity for MHC. Adhesive interactions are not observed at physiological densities of these proteins, and their detection relies on over-expression of either the coreceptor or the MHC molecule (Doyle and Strominger, 1987; Norment et al., 1988). Binding assays also show that soluble CD4 tetramers do not bind detectably to MHC-II-expressing cells, but that very weak binding can be detected when CD4 is coupled to streptavidin-coated beads (∼50,000 CD4s per bead) (Jönsson et al., 2016), emphasizing their profoundly weak binding. T-cell/APC adhesion *in vivo* more likely depends on the much stronger interactions of “professional” adhesion proteins, such as the integrins (Shimaoka et al., 2002) and small adhesion molecules comprising the CD2 subset of the IgSF (Davis et al., 2003). What, then, would be the physiological relevance of a very weak, monovalent interaction if not to increase overall T-cell/APC adhesion? Computer simulations that take into account low affinity CD4/MHC-II interactions suggest that CD4-Lck recruitment would stabilize the TCR/pMHC interaction by only 2–20% and enhance TCR phosphorylation only 3-fold compared to free Lck in the membrane. In contrast, the recruitment of CD4-Lck to a pre-phosphorylated TCR results in a 30- to 40-fold increase in the rate of receptor phosphorylation compared to when CD4-Lck is recruited to an unphosphorylated TCR (Jönsson et al., 2016). On this basis it can be argued that coreceptors significantly enhance antigen-specific signaling only after it is initiated.

### Lck and Coreceptor Occupancy

**FIGURE 2:**
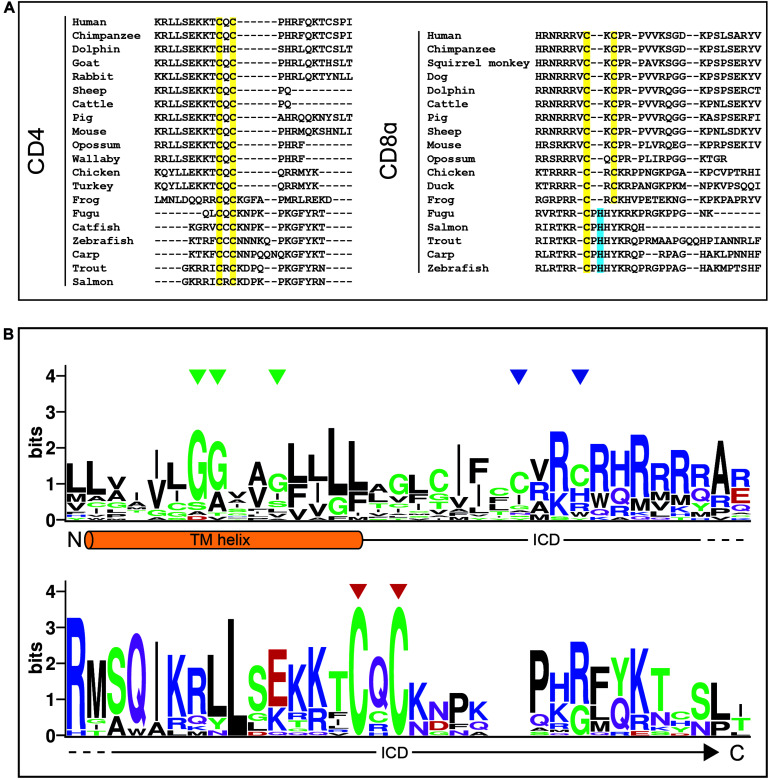
The coreceptor “zinc clasp” is highly conserved. **(A)** MUSCLE alignment of C-terminal CD4 and CD8α sequences with clasp cysteines highlighted in yellow and histidines in equivalent positions highlighted in blue. Adapted from Chida et al. (2011). **(B)** A sequence LOGO of the CD4 transmembrane (TM) helix and intracellular domain (ICD). Green triangles indicate glycines in the conserved GGXXG motif, blue triangles indicate S-palmitoylation sites, and red triangles indicate clasp cysteines. Sheep CD4 was excluded from this analysis due to the presence of large insertions in this regions bearing no homology to any other species.

CD4 and CD8 both associate with Lck via a cytoplasmic “zinc clasp” formed by dicysteine motifs in the coreceptor tail and the Lck SH4 domain (Kim et al., 2003). Lck association is indispensable for coreceptor function as transgenic T cells expressing truncated “tailless” CD4 or CD8αβ molecules have severely diminished responses to *in vitro* stimulation (Zamoyska et al., 1989; Miceli et al., 1991). Supporting this contention, alignment of CD4 and CD8α sequences reveals that the “clasp” cysteines are very highly conserved across vertebrates (Figure 2A, highlighted in yellow). A LOGO analysis of the transmembrane helix and cytoplasmic tail of vertebrate CD4 sequences (Figure 2B) indicates that the “clasp” cysteines are more highly conserved than any other element, including the palmitoylation sites (Crise and Rose, 1992) and the glycine-rich transmembrane region (Parrish et al., 2015). In contrast, the extracellular MHC-binding sites are highly variable (Chida et al., 2011) presumably because the coreceptors had to accommodate a variety of MHC molecules (Sommer, 2005), which allowed diversity in the binding region to emerge. These observations emphasize that Lck association is an ancient and essential feature of coreceptors. Interestingly, all of the CD8α orthologs available from fish species lack the second cysteine residue in the “clasp” motif, where it is replaced where a histidine (Figure 2A, highlighted in blue). Histidine is the second most common Zn^2+^-coordinating residue after cysteine (Dokmanić et al., 2008), reinforcing the notion that CD8α has to associate with Lck.

The coreceptor-Lck interaction was identified in the late 1980s by the Rudd and Schlossman groups who used co-immunoprecipitation (co-IP) assays to show that Lck was linked to CD4 and CD8 in T-cell lysates (Rudd et al., 1988; Barber et al., 1989). Preliminary observations indicated that the fraction of coreceptors associated with cytoplasmic Lck (referred to here as “occupancy”) was high (Veillette et al., 1988) although this was not accurately measured. The first quantitative study was undertaken in the early 1990s by Carmo et al. (1993) who used radioactive antibody fragments to tag CD4. By labeling cell surface CD4 molecules prior to co-IP, the authors could carefully compare the amounts of radioactivity in anti-Lck “pulldowns” relative to anti-CD4 pulldowns, yielding a CD4-Lck occupancy of ∼80% (Carmo et al., 1993). Since this was consistent with the emerging idea that coreceptors recruit kinase activity to the TCR (Rudd et al., 1989; Janeway, 1992), the matter was considered settled. However, several recent studies are beginning to cast doubt on the assumption that coreceptors are wholly occupied by Lck. In 2016 and 2020, two groups reported unexpectedly small occupancy values using the co-IP method: 6% (Parrish et al., 2016) and 37% (Horkova et al., 2020) for the CD4-Lck interaction in single-positive T cells. Even lower values were reported for CD8^+^ T cells and double-positive thymocytes (Horkova et al., 2020). Why would similar assays produce such drastically different occupancy values? One possibility is simply protocol (e.g., incubation periods, different controls, presence or absence of EDTA). Another is that the coreceptor/Lck “clasp” interaction is relatively weak. Assuming typical on-rates [10^5^ M^–1^ s^–1^ (Schlosshauer and Baker, 2004)], the K_d_ values given by isothermal titration calorimetry (Kim et al., 2003) give k_off_ values of 0.04 s^–1^ for CD4-Lck and 0.09 s^–1^ for CD8-Lck, i.e., half-lives of ≈17 s and ≈8 s, respectively. This rapid decay suggests that additional interactions, for example involving the lipid modifications on CD4 (Crise and Rose, 1992), CD8β (Arcaro et al., 2000) and Lck (Paige et al., 1993), make important contributions to complex stability in mixed micelles, and these contributions are difficult to control for. Another limitation of the co-IP method is the risk of sampling interactions in intracellular compartments such as the ER and Golgi, and not just the plasma membrane. High resolution imaging approaches will likely be needed to settle the matter of occupancy, and to ascertain whether the bound and free states of Lck are also modulated as recently proposed (Wei et al., 2020).

## Theories of Coreceptor Contributions to TCR Signaling

### The Lck Recruitment Model

**FIGURE 3:**
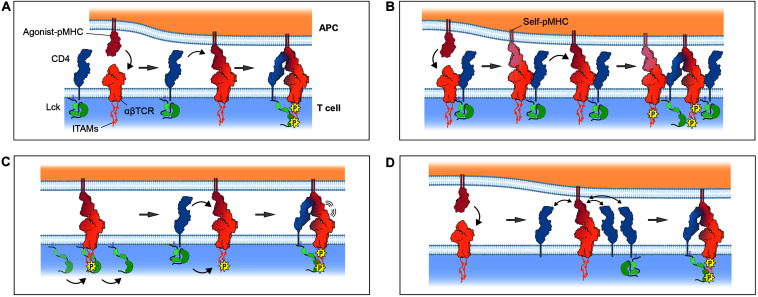
Models for coreceptor function. **(A)** The Lck recruitment model: coreceptors recruit Lck to cognate pMHC-TCR complexes. **(B)** The pseudodimer model: coreceptors cross-link agonist-bound TCR to self-bound TCR. **(C)** The coreceptor recruitment model: ITAMs are incipiently phosphorylated by free Lck prior to recruitment of a coreceptor/Lck complex through SH2 domain-dependent interactions, e.g., between Lck and phosphorylated ITAMs. **(D)** The coreceptor scanning model: a cognate pMHC-TCR complex scans multiple “empty” coreceptors before encountering coreceptor-bound Lck. Gray arrows denote the passage of time. Only the zeta chain ITAMs are shown for simplicity. Protein models were generated from the crystal structures of TCR/CD3 (PDB ID: 6JXR), CD4 (PDB ID: 1WIQ), HLA-DR1 (PDB ID: 4I5B), and the ternary TCR-pMHC-CD4 complex (PDB ID: 3T0E).

The first and simplest proposal for coreceptor function was that CD4 and CD8 have the special role of delivering Lck to the ligand-bound TCR (Figure 3A; Rudd, 1990; Janeway, 1992). This idea incorporated three important experimental observations: (1) the TCR lacked intrinsic kinase activity but was phosphorylated upon ligand engagement (Samelson et al., 1986), (2) coreceptor activity was highest when the MHC- and Lck-binding sites were simultaneously intact (Miceli and Parnes, 1991), and (3) coreceptors became physically associated with the TCR during T-cell activation (Dianzani et al., 1992). A compelling feature of this proposal was that it offered a simple explanation for TCR triggering, since only agonist ligands could form sufficiently stable TCR/pMHC complexes to permit coreceptor recruitment and TCR phosphorylation. However, it was later shown that TCR signaling could be coreceptor-independent (Locksley et al., 1993; Schilham et al., 1993) indicating that CD4 and CD8 are not essential for triggering. A prediction of the Lck recruitment model was that soluble pMHC monomers would trigger signaling, but this is now known not to occur (Boniface et al., 1998; Schott et al., 2002). Finally, given the poor ability of MHC-II molecules to recruit CD4 to the TCR (Jönsson et al., 2016), these observations indicate that the role of coreceptors is not to trigger *de novo* signaling by recruiting Lck to the TCR/pMHC complexes.

### The Pseudodimer Model

When the structures of coreceptor-MHC complexes were first solved it was found, somewhat surprisingly, that coreceptors engage membrane-proximal regions of MHC molecules almost directly orthogonal to the TCR-binding site (Gao et al., 1997; Wang et al., 2001). The resulting topology was expected to prevent the TCR and the coreceptor from physically associating (van der Merwe and Davis, 2003), whereas it had been proposed that such an association could be important for signaling. One idea that could reconcile these arguments was the “pseudodimer” model (Figure 3B; Irvine et al., 2002) in which coreceptors are thought to bridge the gap between adjacent TCRs to form receptor pseudodimers. According to this idea, an agonist-bound TCR is stabilized by the recruitment of a second self-pMHC-bound TCR through a cross-linking coreceptor to create a geometry permissive for Lck-mediated phosphorylation (Krogsgaard et al., 2005). The main advantage of this hypothesis was that it provided an explanation for the observation that soluble, covalently linked pMHC dimers, consisting of an agonist-pMHC and a self-pMHC, could be shown to induce T-cell activation (Krogsgaard et al., 2007). However, structural studies offer little support for the notion that either affinity-matured (Yin et al., 2012) or native (Jönsson et al., 2016) CD4 associates physically with the TCR in the orientation required for pseudodimerization. This is less clear for CD8 because no structural information is available for the stalk region of the protein. However, glycosylation is thought to make the stalk rigid (Li et al., 2013) which could prevent CD8 bridging two TCRs. A second issue is that this idea, again, runs into the problem of the very low coreceptor/MHC affinity. Pseudodimerization, relying as it does on the interaction of the coreceptor with MHC proteins only, is unlikely to produce the levels of signaling enhancement typically observed in the presence of CD4 or CD8.

### The Two-Step Coreceptor Recruitment Model

Xu and Littman introduced a new heterodoxy in 1993, proposing that the “delivery of a catalytically active Lck to the TCR complex is not the primary function of CD4.” They suggested instead that the coreceptor function was modulated by TCR triggering, and not *vice versa*. In a series of remarkable experiments, Xu and Littman (1993) showed that the activity of a CD4-Lck chimera was (1) *abolished* by mutating its phosphotyrosine-binding SH2 domain, and (2) *increased* by deleting its kinase domain. Both results pointed to the dominant role not of the kinase domain of Lck in enhancing TCR triggering, but rather its SH2 domain. Xu and Littman (1993) interpreted the first of these results as implying that coreceptor function depended crucially on prior phosphorylation of the TCR. They proposed that the second, even more troubling, of these observations could be explained by the absence of the C-terminal tyrosine of the kinase domain, which would normally become phosphorylated and block SH2 domain access. In support of these interpretations, biochemical studies had by then shown how TCR triggering enhances the binding of CD4 to the TCR (Mittler et al., 1989) and CD8 to MHC class I (O’Rourke et al., 1990).

Xu and Littman (1993) proposed a radical new, two-step mechanism of TCR triggering later referred to as the “coreceptor recruitment model” (van der Merwe and Cordoba, 2011; Figure 3C): (1) the ligand-engaged TCR complex is initially and partially phosphorylated by free Lck diffusing in the membrane, followed by (2) the recruitment of a coreceptor/Lck complex to the engaged TCR/pMHC, via bidentate interactions between the extracellular regions of the coreceptor and pMHC, and between the SH2 domain of Lck and phosphotyrosines in the cytoplasmic regions of the TCR. Direct evidence for a two-step process did not emerge until 2011, however, when Jiang et al., 2011 observed it directly using a bespoke mechanical adhesion frequency assay. By repeatedly bringing CD8^+^ T cells into contact with red blood cells (RBCs) used as surrogate APCs and measuring the resulting RBC membrane deformation, Jiang et al. (2011) observed that bonds formed more frequently than expected for the simple sum of TCR/pMHC and pMHC/CD8 bonds, which was indicative of cooperative binding. They went on to show that the cooperative binding was induced and that it was blocked by kinase inhibitors. This data was interpreted as offering strong support for the proposal of Xu and Littman (Jiang et al., 2011; van der Merwe and Cordoba, 2011). Similar support for TCR-CD8 cooperation in binding pMHC were obtained for human T cells recognizing self-antigens (Liu et al., 2014). A potential mechanism for this two-step model was identified in 2014 when Gascoigne and colleagues, using FRET measurements, showed that free, coreceptor-unbound Lck catalyzes the initial phosphorylation of the ligand-engaged TCR, and that CD8 recruitment depends critically on the CD8-Lck “clasp” interaction (Casas et al., 2014).

But how do CD4 and CD8 increase T-cell sensitivity if TCR signaling and T-cell activation are not necessarily coreceptor-dependent (Locksley et al., 1993; Schilham et al., 1993)? These observations could be reconciled if it is first postulated that initial phosphorylation of the TCR is catalyzed inefficiently by free Lck but, in certain circumstances, e.g., for high affinity TCRs, this is enough to activate a cell. A second requirement would be that levels of incipient phosphorylation are sufficient for coreceptor recruitment, which then increases the initial signal. But how would enhanced signaling arise? Xu and Littman (1993) suggested that coreceptors contribute to the formation of a stable ternary signaling complexes and amplify an initially low level of TCR phosphorylation via the catalytic activity of Lck. Modeling studies are consistent with the second of these ideas and show that both coreceptors act primarily to shuttle Lck to the TCR (Li et al., 2004; Artyomov et al., 2010). But imaging and biophysical experiments show that CD4 has a negligible effect on the affinity and lifetime of TCR-pMHC complexes *in situ* (Huppa et al., 2010; Hong et al., 2015), although there is some evidence that CD8 has an additional contribution to complex stabilization (Wooldridge et al., 2005; Jiang et al., 2011). Importantly, the two-step coreceptor recruitment mechanism may ensure that coreceptor-mediated signal amplification is subservient to primary agonistic TCR signaling (Davis et al., 2003).

The ability of CD8, but not CD4, to increase the stability of the ternary complex has been attributed to the greater affinity of CD8 for MHC molecules (Artyomov et al., 2010). This raises the question of why CD4 and CD8 have such different affinities for MHC given their highly analogous functions. It might be that the contribution of each coreceptor is “tuned” to the physiological context in which they function. For example, CD8 may have to bind strongly to MHC-I because the targets of CD8^+^ cytotoxic T lymphocytes (CTLs) are often infected or malignant somatic cells that do not express co-stimulatory molecules such as CD80/CD86 (McAdam et al., 1998), in contrast to the targets of CD4^+^ helper T (Th) cells.

## Coreceptors and the Thymus

### Thymic Development

Coreceptors are important for coupling the two principal T-cell effector functions of “help” and “killing” to MHC class. They ensure that, in the periphery, CD4^+^ T-cells are only activated by pMHC-II on professional APCs while CD8^+^ T-cells can respond to foreign or mutated peptides on all MHC-I-expressing somatic cells. This dichotomy is established during a complex developmental program in the thymus. Developing thymocytes express randomly generated TCRs that are tested against self-pMHC molecules. Weakly self-reactive thymocytes receive a survival signal, producing a pool of cells capable of recognizing host MHC proteins (positive selection), whereas strongly self-reactive thymocytes are deleted to avoid autoreactivity (negative selection). In addition, a limited number of strongly self-reactive thymocytes develop into regulatory T-cells that suppress harmful autoimmune responses and inflammation. Together, these processes drive the generation of mature, peripheral T-cells which are appropriately self-MHC-restricted and self-tolerant.

CD4 and CD8 are critically important for the maturation of MHC-restricted T cells, as illustrated by the failure of CD4- and CD8-deficient mice to generate CD4^+^ Th or CD8^+^ CTLs, respectively (Fung-Leung et al., 1991; Rahemtulla et al., 1991). But Lck itself must also play a central role since the simultaneous deletion of Lck and a closely related kinase called Fyn results in a complete failure to produce αβ T cells (van Oers et al., 1996). In 2007, Singer and colleagues proposed that CD4 and CD8 confer MHC restriction on developing T cells by sequestering Lck away from TCRs that, by chance, engage non-MHC thymic ligands that cannot also interact with the coreceptors (Van Laethem et al., 2007). According to Xu and Littman’s (1993) two-step signaling mechanism, however, T cells encountering these ligands would be expected to develop if their TCRs bound strongly enough for free Lck to produce sufficient signaling to negotiate positive and negative selection. These predictions were borne out when Van Laethem et al., 2007 showed that mice lacking CD4, CD8, MHC-I and MHC-II (so called “quad-deficient” mice) produced a diverse repertoire of αβTCR-expressing, MHC-unrestricted T cells. Singer and colleagues then established the binding specificities of two such TCRs, and found that the TCRs bound the surface protein CD155 in a manner similar to antibodies, i.e., to distinct conformational epitopes, with nanomolar affinity, and without any involvement of MHC proteins (Tikhonova et al., 2012; Lu et al., 2020). Whether or not MHC selection in the thymus is entirely dependent on Lck sequestration by the coreceptors, or evolutionary pressures on the germline have also encoded a set of “rules of engagement,” will likely continue to be debated (Garcia, 2012; Van Laethem et al., 2012). But how would naturally occurring MHC-unrestricted T cells develop if signaling is normally coreceptor-dependent? The Singer group have also shown that the timing of coreceptor expression is carefully controlled allowing, for example, signaling in γδTCR-expressing thymocytes to be triggered by free Lck before CD4 or CD8 are expressed (Van Laethem et al., 2013).

### Coreceptor Scanning as a Discriminative Mechanism

Signaling by the TCR needs to reach sufficient levels to activate a mature T cell, but also be sensitive to the “quality” of a ligand, especially in the service of thymocytes that must discriminate between the self-ligands mediating positive and negative selection. Palmer and colleagues have proposed a role in this for coreceptors in the form of a processive “coreceptor scanning” mechanism (Figure 3D; Stepanek et al., 2014). They suggest that TCR-pMHC complexes would likely have to rapidly engage or “scan” several CD4/CD8 proteins before encountering Lck, because coreceptor occupancy is very low (less than 10%) according to their measurements. The delay between pMHC binding and Lck recruitment, they argued, would allow the TCR to translate small differences in affinity into large differences in response, providing a long sought-after explanation for kinetic proofreading in T-cell activation (McKeithan, 1995). “Coreceptor scanning” is reminiscent of an earlier signaling mechanism called the “occupancy model” in which Lck activity is regulated by altering coreceptor occupancy (Rudd, 1990). However, these mechanisms are effectively refinements of the Lck recruitment model, and therefore suffer from the same general problems as this theory. First, the very low affinity of coreceptor/MHC interactions means that the recruitment of coreceptor-bound Lck, regardless of occupancy, would already be very inefficient. Second, Lck is generally expressed in excess of either CD4 or CD8 (Takada and Engleman, 1987; Davis et al., 1998; Hui et al., 2017; Voisinne et al., 2019; Felce et al., 2020) and the free Lck would be expected to work against low coreceptor occupancy unless it is actively kept low. A third problem is that the work of Xu and Littman (1993) shows that Lck recruitment is dependent on its SH2 domain, i.e., that it requires prior TCR phosphorylation, making discrimination at the point of coreceptor recruitment redundant. Finally, it is unclear how discrimination would be protected from physiological variations in coreceptor expression levels (Itoh et al., 2005; Xiao et al., 2007) which might affect the coreceptor/Lck coupling equilibrium.

How might T cells or thymocytes discriminate between agonists and non-agonists if not through kinetic proofreading mechanisms such as “coreceptor scanning”? One proposal is mechanotransduction, in which the TCR and the coreceptor together are intrinsically capable of differentiating between ligands on the basis of the type of molecular bonds they form (Hong et al., 2018). Another is that antigen discrimination is an emergent property of a signaling mechanism constrained by T-cell topography, i.e., one relying only on receptor dwell-time at phosphatase-depleted regions of contact between T cells and APCs (Fernandes et al., 2019).

## Conclusion

The marking of distinct T-cell subsets by CD4 and CD8 thrust the coreceptors into the limelight from the very outset. But it is now more than 40 years since their special status became apparent, first as coreceptors forming ternary complexes with the TCR and pMHC strongly enhancing T-cell responses (Janeway, 1988, 1992), and then as potent amplifiers of signaling, acting secondarily to TCR triggering (Xu and Littman, 1993). In the ensuing period we have learnt a great deal about the structures and interactions of CD4 and CD8, but there are still many unanswered questions in this area of T-cell biology, including:

1. What is the true occupancy level of coreceptors and does it change during thymic development or between T-cell subsets?
2. Do we fully understand why coreceptor/MHC interactions are so unusually weak? And are there physiologically relevant situations in which they are enhanced (Owen et al., 2016)?
3. Once the bidentate binding to TCR/pMHC is established, what is the mechanism by which coreceptors enable orders-of-magnitude signal amplification?
4. Why are CD4 and CD8 palmitoylated and is it linked to membrane heterogeneity?
5. Are CD4 and CD8 organized within microvilli, microclusters and the corolla or are they randomly distributed across the cell surface, and how does this change in the course of activation?
6. Finally, do CD4 and CD8 play a role in the numerous other leukocytes in which they are expressed (Gibbings and Befus, 2009; Kadivar et al., 2016)?

## Author Contributions

AM wrote the initial draft and generated sequence alignments. All authors made a substantial, direct and intellectual contribution to the work, and approved it for publication.

## Conflict of Interest

The authors declare that the research was conducted in the absence of any commercial or financial relationships that could be construed as a potential conflict of interest.
